# Safety of COVID-19 Vaccination in Pregnancy: A Systematic Review

**DOI:** 10.3390/diagnostics14161775

**Published:** 2024-08-14

**Authors:** Angeliki Gerede, Georgios Daskalakis, Themistoklis Mikos, Christos Chatzakis, Eleftherios Vavoulidis, Makarios Eleftheriades, Ekaterini Domali, Konstantinos Nikolettos, Efthymios Oikonomou, Panagiotis Antsaklis, Marianna Theodora, Alexandros Psarris, Chrysoula Margioula-Siarkou, Stamatios Petousis, Sofoklis Stavros, Anastasios Potiris, Apostolos Athanasiadis, Konstantinos Dinas, Panagiotis Tsikouras, Nikolaos Nikolettos, Alexandros Sotiriadis

**Affiliations:** 1 Unit of Maternal-Fetal-Medicine, Department of Obstetrics and Gynecology, Medical School, Democritus University of Thrake, GR-68100 Alexandroupolis, Greece; k.nikolettos@yahoo.gr (K.N.); eftoikonomou@outlook.com (E.O.); ptsikour@med.duth.gr (P.T.); nnikolet@med.duth.gr (N.N.); 2 First Department of Obstetrics and Gynecology, Medical School, National and Kapodistrian University of Athens, GR-11528 Athens, Greece; gdaskalakis@yahoo.com (G.D.); kdomali@yahoo.fr (E.D.); panosant@gmail.com (P.A.); martheodr@med.uoa.gr (M.T.); sfstavrou@med.uoa.gr (S.S.); apotiris@med.uoa.gr (A.P.); 3 First Department of Obstetrics and Gynecology, Medical School, Aristotle University of Thessaloniki, GR-54124 Thessaloniki, Greece; themismikos@auth.gr; 4 Second Department of Obstetrics and Gynecology, Medical School, Aristotle University of Thessaloniki, GR-54640 Thessaloniki, Greece; cchatzakis@gmail.com (C.C.); e.vavoulidis@yahoo.com (E.V.); margioulasiarkouc@gmail.com (C.M.-S.); petousisstamatios@gmail.com (S.P.); konstantinosdinas@hotmail.com (K.D.); asotir@gmail.com (A.S.); 5 Second Department of Obstetrics and Gynecology, Medical School, National and Kapodistrian University of Athens, GR-11528 Athens, Greece; makarios@hotmail.co.uk; 6 Third Department of Obstetrics and Gynecology, Medical School, Aristotle University of Thessaloniki, GR-54640 Thessaloniki, Greece; apathana@auth.gr

**Keywords:** safety, COVID-19, vaccination, pregnancy

## Abstract

The COVID-19 pandemic has posed significant risks to pregnant women and those recently pregnant, leading to heightened mortality and morbidity rates. Vaccination has emerged as a pivotal strategy in reducing COVID-19-related deaths and illnesses worldwide. However, the initial exclusion of pregnant individuals from most clinical trials raised concerns about vaccine safety in this population, contributing to vaccine hesitancy. This review aims to consolidate the existing literature to assess the safety and efficacy of COVID-19 vaccination in pregnant populations and neonatal outcomes. Diverse studies were included evaluating various aspects of safety for women and their newborns, encompassing mild to severe symptoms across different vaccines. The findings indicate the overall safety and efficacy of COVID-19 vaccination, with minimal adverse outcomes observed, including mild side effects like pain and fever. Although most studies reported the absence of severe adverse outcomes, isolated case reports have raised concerns about potential associations between maternal COVID-19 vaccination and conditions such as fetal supraventricular tachycardia and immune-mediated diseases. Our review underscores the importance of ongoing surveillance and monitoring to ensure vaccine safety in pregnant women. Overall, COVID-19 vaccination during pregnancy remains a safe and effective strategy, emphasizing the need for continued research and vigilance to safeguard maternal and fetal health.

## 1. Introduction

Global health data have consistently shown since the beginning of the COVID-19 pandemic that pregnant women and those who have recently been pregnant and become infected with SARS-CoV-2 are at a heightened risk of experiencing severe COVID-19 symptoms, as well as increased mortality and morbidity [1,2]. In the ongoing fight against the pandemic, vaccination has emerged as the primary pillar of preventive strategies, significantly reducing COVID-19-related deaths and illnesses across various populations [3,4]. However, a critical gap existed regarding the safety of COVID-19 vaccines for pregnant women since most phase III trials excluded them [5]. This knowledge gap led to a complex dilemma in the pregnant population. While recommendations strongly advocated for pregnant individuals to receive the COVID-19 vaccine, concerns about potential risks to maternal and fetal health had contributed to vaccine hesitancy among this group [5,6]. This hesitancy was also amplified by the novel molecular technologies employed in many COVID-19 vaccines, which differed from the conventional methods used in vaccine construction prior to the pandemic [7]. Additionally, concerns were heightened by the comparatively short time frame in which these vaccines were developed [8].

Fortunately, cohort studies conducted in the past few years have provided valuable insights into the safety and efficacy of the vaccine in pregnant women and fetal health. The aim of this review was to consolidate the existing literature and assess whether COVID-19 vaccination is safe for use in the pregnant population including studies conducted in diverse populations that received different types and doses of vaccines while evaluating various aspects of safety for women and their newborns, from mild to severe symptoms. A summary of studies that have evaluated the safety of eight different COVID-19 vaccine brands, namely BNT162b2 (Pfizer-BioNTech, New York, NY, USA), mRNA-1273 (Moderna, Cambridge, MA, USA), Covishield (AstraZeneca, Cambridge, UK), Janssen (Johnson & Johnson, New Brunswick, NJ, USA), CoronaVac (Sinovac, Sheung Wan, Hong Kong), Covax (Sinopharm, Shanghai, China), Sputnik V (RDIF, Moscow, Russia), Covaxin (Bharat Biotech, Hyderabad, India). It is worth noting that many of the studies focused on populations who received mRNA vaccines such as Pfizer-BioNTech and Moderna. Among the adverse outcomes assessed in the studies included in this review were immunogenicity, frequency of miscarriages, genetic and non-genetic congenital anomalies in newborns, birth weight, rate of cesarean section, and mild side effects like pain and fever. Additionally, case studies reporting specific conditions related to vaccination were incorporated to provide an overview of potential risks.

The primary aim of this comprehensive review is to thoroughly examine and synthesize the available data and evidence on the safety and efficacy of COVID-19 vaccines for pregnant individuals. By delving into the existing literature, this study seeks to inform and educate the scientific community, providing them with a valuable resource on this important topic. Additionally, the insights and recommendations generated through this analysis are intended to serve as a guide for healthcare providers and policymakers, empowering them to make informed decisions regarding vaccination strategies for pregnant individuals. Through a meticulous and detailed assessment of the current research, this review strives to offer actionable insights that can help optimize COVID-19 vaccination programs and ultimately safeguard the health and well-being of pregnant women and their unborn children.

## 2. Materials and Methods

Study design: Employing a systematic and exhaustive methodology, this study aimed to evaluate the safety of COVID-19 vaccination in pregnancy. A thorough exploration of the literature was conducted to locate relevant studies, followed by a stringent selection process based on predetermined inclusion and exclusion criteria.

Literature search: The databases Scopus and PubMed were searched to identify relevant studies using keywords including <<COVID-19 or SARS-CoV-2 or SARS coronavirus 2) and (vaccination or vaccine) and (pregnancy or gestation) and safety>>. The search algorithm was adjusted for each database while maintaining a common overall architecture.

Studies selection and eligibility: This review incorporated observational studies, cohort studies, and descriptive and case studies, documenting the effects of any COVID-19 vaccine administered to women before or during pregnancy, along with maternal and offspring outcomes related to pregnancy. The studies were sourced up to April 2024. We omitted studies lacking pregnant populations, as well as review articles, meta-analyses, and studies not published in English.

This review was performed in accordance with the PRISMA 2020 (Preferred Reporting Items for Systematic Reviews and Meta-Analyses) guidelines (the review was not registered) [9] (Table S1). After the initial literature search, two independent authors screened them for relevance based on titles and abstracts only. Disagreements were resolved through consensus or by discussion with a third author. Articles deemed as irrelevant were excluded and the full-text copies of the remaining articles were assessed for eligibility as per the PICOS criteria by two blinded reviewers. Inconsistencies were, once again, resolved by consensus or by a third reviewer. The references of the full-text copies were accessed to prevent the potential loss of eligible studies that were missed by the database search (snowball procedure). Any discrepancies between the reviewers were resolved through discussion with a third reviewer, resulting in no relevant studies being excluded). The following data items were extracted from the eligible studies; year of publication, study design, country, center and time period during which the study was conducted, number of participants, age, type of COVID-19 vaccination, immunogenicity effects, general adverse outcomes, miscarriages, booster doses and specific medical conditions.

## 3. Results

### 3.1. The Selection Process of Included Studies

**Figure 1:**
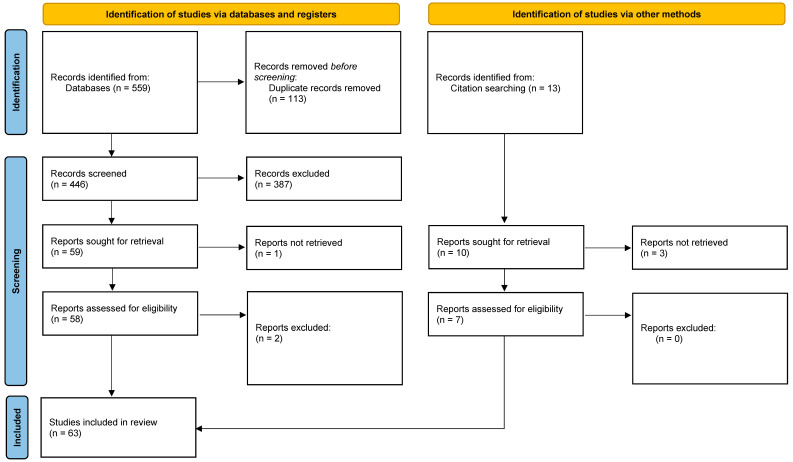
Flow diagram of the included studies.

A flow diagram of the selection process is presented in Figure 1. In total, 559 papers were initially identified, and after duplicate removal, 446 were considered eligible for title–abstract screening. Subsequently, 58 articles were selected for full-text screening; all of which met the inclusion criteria and were included in this review. Furthermore, the references of the included studies and references from other relevant studies from high-impact journals were hand-searched and seven papers that were lost from the initial literature search were included as well. Thus, the 63 studies that were included in total investigated the association between. A summary of the included study characteristics is provided in Table 1.

### 3.2. COVID-19 Vaccines

Before embarking on studies assessing the safety of COVID-19 vaccination in pregnant populations, it is crucial to understand the mechanisms of action of the current vaccines available. Understanding the molecular technologies used to construct COVID-19 vaccines is particularly important because this new approach to vaccine development led to hesitancy among many individuals. Moreover, comprehending these differences is vital for identifying potential risks they may pose. This knowledge empowers healthcare providers and individuals to make informed decisions, weighing the benefits and risks of vaccination during pregnancy.

Traditional vaccines can be categorized into three types: live, inactivated, and component vaccines. Live vaccines use weakened viruses or bacteria, while inactivated vaccines contain killed pathogens [73]. Component vaccines use purified or chemically synthesized antigens [73]. However, the current COVID-19 vaccines employ newer molecular technologies [74,75]. For instance, Pfizer-BioNTech and Moderna vaccines contain mRNA-encoding viral spike proteins enclosed in lipid nanoparticles. The AstraZeneca vaccine contains fragments of SARS-CoV-2 cDNA in an adenovirus vector. When administered, these vaccines are taken up by immune cells and trigger an immune response, leading to the production of neutralizing antibodies and cytotoxic T cells. Different vaccines elicit varying immune responses; AstraZeneca and Pfizer-BioNTech vaccines stimulate cellular immune responses mediated by specific cells while other COVID-19 vaccines, such as Sinovac and Sinopharm, utilize inactivated viruses, while Sputnik uses adenovirus vectors [73]. There are additional reviews available in the literature that delve deeper into this subject [8,76].

### 3.3. Immunogenicity

Exploring the efficacy of COVID-19 vaccines in pregnant women began with a focus on their immunogenicity, marking a critical early investigation into their effectiveness within this specific demographic. Studies already from 2021 investigated the effectiveness of the BNT162b2 mRNA vaccine in pregnant women, revealing its capability to elicit a humoral immune response, albeit with slightly lower SARS-CoV-2 IgG levels compared to non-pregnant counterparts [17]. In 2022, further research delved into the impact of COVID-19 infection or vaccination during pregnancy on the decidual immune environment [45]. Findings indicated no significant differences in decidual immune cells or cytokines between COVID-19 infected and control groups, while vaccinated cohorts exhibited lower levels of certain immune cells without inflammation. Heterologous vaccination also emerged as a viable option, inducing effective immunogenicity, and offering an alternative in mRNA-limited settings [19]. Furthermore, investigations into mRNA-1273 and BNT162b2 vaccines extended to lactating women, revealing immunogenicity and the transfer of vaccine-elicited antibodies to infant cord blood and breast milk in both pregnant and non-pregnant cohorts [21].

### 3.4. General Adverse Outcomes

Theiler et al. reported that vaccinated pregnant women were less likely to become infected with COVID-19 and did not experience increased pregnancy or delivery complications compared to unvaccinated individuals [63]. Meanwhile, another study compared short-term outcomes, like vaginal bleeding, pregnancy loss, hypertension, and gestational diabetes, in vaccinated vs. non-vaccinated pregnant women and found no significant differences [15]. In a cohort study by Kachikis et al., the experiences of pregnant and lactating individuals after receiving COVID-19 vaccines were investigated, highlighting common reactions such as pain at the injection site and fatigue [46]. Some participants reported interrupted breastfeeding and decreased milk supply among lactating individuals.

Jorgensen et al. conducted a population-based retrospective cohort study, which revealed that infants born to vaccinated mothers exhibited lower risks of severe neonatal morbidity, neonatal death, and admission to the neonatal intensive care unit [44]. Similarly, Tavares et al. provided evidence that vaccination during all trimesters of pregnancy, regardless of the vaccine type, did not increase the risk of adverse birth outcomes or neonatal deaths [62].

Retrospective cohort studies by Fell et al. indicated that COVID-19 vaccination during pregnancy was not associated with increased risks of adverse peripartum outcomes or adverse pregnancy outcomes, supporting its safety [10,32]. Additionally, a study by Smithgall et al. on placental pathology [77] and an evaluation by Shanes et al. of key placental lesions [78] found no significant differences between vaccinated and unvaccinated groups, further affirming the safety of vaccination during pregnancy. Moreover, vaccinated women showed fewer maternal and fetal vascular malperfusion placental features, suggesting the vaccine’s protective effect in COVID-19-infected pregnant patients [61].

Findings from the study by Hatami et al. offer additional support for inactivated COVID-19 vaccines, indicating no adverse pregnancy outcomes associated with this vaccination type during the second and third trimesters [42]. Yang et al. and Li et al. focused on evaluating the safety of inactivated COVID-19 vaccines administered during the peri-pregnancy period [50,79]. A study by Yang et al. reported no significant differences in maternal premature rupture of membranes or neonatal adverse events between the vaccine and control groups. However, there was a notable difference in serum alanine transaminase (ALT) levels during the first trimester, with higher levels observed in the vaccine group. Despite this, the study concludes that inactivated COVID-19 vaccination during the peri-pregnancy period is safe for both pregnant women and neonates, regardless of the timing of vaccination or medication use. Additionally, it recommends monitoring ALT levels throughout the first trimester of pregnancy [50]. A study by Li et al. primarily focused on neonatal malformations, with secondary indicators including adverse pregnancy and delivery events, fetal development metrics, and various pregnancy complications. Key findings include no increase in neonatal congenital abnormalities among vaccinated women, and similar neonatal length, weight, head circumference, and incidence of asphyxia in both vaccinated and unvaccinated groups. However, there was a higher incidence of neonatal jaundice in the vaccinated group, with maternal peri-pregnancy vaccination identified as an independent risk factor [79].

Expanding on the safety of mRNA vaccines, studies indicate that these vaccines are generally safe in pregnant women across trimesters [12], with maternal vaccination reducing the risk of infant hospitalization due to COVID-19, underscoring the protective benefits for newborns [40]. It was shown that BNT162b2 vaccination was not associated with adverse outcomes in pregnant women and significantly lowered the risk of SARS-CoV-2 infection compared to unvaccinated pregnant women, highlighting the protective benefits of vaccination in this population [35]. Additionally, the same author in a different study [36] found that women vaccinated with mRNA vaccines during pregnancy did not experience higher adverse pregnancy or neonatal outcomes compared to historical background risks in the obstetric population. Building upon these findings, Boelig et al. observed inflammatory effects associated with COVID-19 but not with vaccination and provided pathophysiological and clinical evidence supporting the safety of mRNA vaccines in pregnancy [16]. Similarly, Norman et al. found no heightened risks of adverse neonatal events in infants born to mRNA-vaccinated mothers [56]. It is also worth noting the results of an in vitro study that assessed the impact of Pfizer-BioNTech mRNA vaccination on the development of syncytiotrophoblast (STB), a crucial cell layer of the placenta [66].

More recently, in a robust analysis encompassing a vast cohort of over 300,000 pregnant individuals, Faherty et al. found no discernible links between COVID-19 vaccination and adverse outcomes such as preterm birth or stillbirth [31]. Collectively, the findings of these studies provide robust support for the safety of COVID-19 vaccination during pregnancy.

### 3.5. Special Adverse Outcomes

#### 3.5.1. Miscarriages

Miscarriage, also known as spontaneous abortion, refers to the loss of the fetus that occurs within the first 22 weeks of pregnancy. Various factors, including genetic abnormalities, hormonal issues, maternal health conditions, or infections, like COVID-19 can lead to miscarriage. COVID-19 vaccination in pregnant women has been suggested as a strategy to mitigate the risk of miscarriages. However, concerns have been raised regarding whether the vaccine itself could potentially trigger miscarriages. This question was addressed by Velez et al., and De Feijter, whose research indicated that SARS-CoV-2 vaccination did not correlate with an increased risk of miscarriages. Their findings support the safety of COVID-19 vaccine during pregnancy [23,68].

Similarly, in a study conducted in 2022, researchers investigated the extent to which preconception maternal or paternal COVID-19 vaccination is linked to miscarriage incidence [80]. This study was the first to prospectively evaluate the relationship between preconception COVID-19 vaccination in both partners and miscarriage. The results concluded that COVID-19 vaccination in either partner at any time before conception is not associated with an increased rate of miscarriage. Furthermore, Mascolo et al. demonstrated that there was no significant association between spontaneous abortion and BNT162b2, mRNA-1273, ChAdOx1-S/nCoV-19, or mixed vaccination observed in pregnant women [55].

Another noteworthy, matched cohort study examined the connections between COVID-19 vaccination and miscarriage before 20 weeks of gestation, as well as ectopic pregnancy [18]. This study revealed no association between vaccination with BNT162b2, mRNA-1273, or ChAdOx1-S/nCoV-19 vaccines and either miscarriage or ectopic pregnancy. These findings align with those of Trostle et al., although their study focused solely on women vaccinated with mRNA vaccines and lacked a matched control group [65].

Expanding on this data, the studies by Mansour et al. and Citu et al. indicate no significant association between either version of the mRNA vaccine and miscarriage rates [20,54]. Gastesi et al. demonstrated that there is no increased risk of miscarriages associated with women vaccinated with two doses of the COVID-19 vaccine [33]. However, in this study, a trend towards an increased risk of miscarriage was observed among women who received only one dose. The results related to receiving two doses may be influenced by selection bias, specifically “survival bias”, potentially diluting the true effect. Overall, the authors suggest interpreting these results cautiously due to low numbers, emphasizing the need for further studies to clarify the true association. Lastly, a valuable finding from the study by Vazquez et al. revealed that the timing of surveillance appears to be an important factor affecting the observed association between vaccination and spontaneous abortion [67].

#### 3.5.2. Congenital Anomalies

The risk of congenital anomalies associated with COVID-19 vaccination has been explored in a limited number of studies. Congenital anomalies are structural or functional abnormalities present at birth, which can affect various parts of the body such as organs, limbs, or systems. A national, population-based, matched cohort study led by Calvert et al. examined the impact of mRNA vaccines on the risk of major congenital anomalies during gestation [18]. Their investigation revealed no significant association between COVID-19 vaccination and the occurrence of either any major congenital anomaly or any non-genetic congenital anomaly. However, it should be also mentioned that no notable link was found between SARS-CoV-2 infection during gestation and the presence of congenital anomalies. These findings provide valuable insights into the safety of mRNA vaccines in pregnancy concerning congenital anomalies. In another cohort study investigating the association between COVID-19 vaccination during the first trimester and the occurrence of major non-genetic congenital anomalies in offspring, reassuring findings emerged [72]. The study found no indications of an elevated risk of major non-genetic congenital anomalies in offspring following maternal COVID-19 vaccination during the first trimester. The findings of these two studies, provide valuable insights into the safety of mRNA vaccines in pregnancy concerning congenital anomalies.

### 3.6. Booster Doses

The available data on booster doses of vaccination are also crucial for evaluating their efficacy and safety profile in pregnant women. Dick et al. investigated the impact of receiving a booster dose of the SARS-CoV-2 vaccine during pregnancy on obstetrical outcomes [28]. Primary outcomes focused on the incidence of preterm labor and small for gestational age neonates, while secondary outcomes included other maternal and neonatal complications. Results showed no association between receiving the booster dose during pregnancy and adverse obstetrical outcomes compared to unvaccinated or twice-vaccinated women. However, higher rates of postpartum hemorrhage were noted. Similarly, the findings from Toussia et al. provide assurance regarding the safety profile and immunogenicity of the BNT162b2 vaccine’s second and third doses among pregnant women [64]. Notably, the third dose exhibits efficacy in eliciting a more robust humoral immune response in pregnant individuals compared to the second dose, without evidence of early obstetric complications. Furthermore, another study offers reassurance regarding the safety of administering the third BNT162b2 mRNA COVID-19 vaccination dose during pregnancy [39]. It is also noteworthy that receiving a COVID-19 booster vaccination during pregnancy is not associated with an increased risk of spontaneous abortion within 42 days, regardless of whether it was a third mRNA vaccine dose or any COVID-19 vaccine booster [47].

Studies have also highlighted the benefits of COVID-19 booster vaccination during pregnancy. Individuals who receive booster doses exhibit heightened protection [58]. Additionally, it has been reported that administering a booster dose during pregnancy significantly increases both maternal and cord blood binding and neutralizing antibody levels, offering substantial defense even against emerging variants such as Omicron BA.1 [47]. Furthermore, administering mRNA monovalent COVID-19 booster vaccinations during pregnancy was found not to increase the risk of serious adverse events such as thrombocytopenia, myocarditis, venous thromboembolism, or ischemic stroke within either a 21 or 42-day period post-vaccination. However, there was an observed higher likelihood of experiencing medically attended malaise or fatigue within 7 days, as well as lymphadenopathy or lymphadenitis within 21 days after vaccination [26].

### 3.7. Specific Medical Conditions

Lastly, it is imperative to consolidate various case reports and studies to elucidate potential associations between COVID-19 vaccination and specific medical conditions or adverse outcomes. Thoroughly reviewing these data provides valuable insights into the global impact of vaccines on the pregnant population.

A case study by Abdallah et al. raises concerns about a potential link between maternal COVID-19 vaccination, specifically with the Pfizer-BioNTech vaccine, and fetal supraventricular tachycardia (SVT) [11]. This study documents the first two reported cases of fetal SVT following maternal administration of the mRNA vaccine. While there were no previous reports of such occurrences, the study highlights those vaccines can induce tachycardia, with cardiac arrhythmia being a possible side effect. The study emphasizes the importance of ongoing safety monitoring and longitudinal follow-up to better understand the fetal impact following maternal COVID-19 vaccination. Additionally, adverse events related to COVID-19 vaccination in pregnant women with systemic lupus erythematosus (SLE) have been examined through a global e-survey. Despite the limited sample size, the study sheds light on the safety of COVID-19 vaccines in this specific population during pregnancy and lactation [34].

A case report by Rysava et al. discusses a rare but serious condition called atypical hemolytic uremic syndrome (aHUS), which is characterized by dysregulation in the alternative complement activation pathway [60]. Typically, it leads to acute kidney injury and organ ischemia, often triggered by various factors such as infections, pregnancy, surgery, and injuries. The report presents a case of a young woman with a history of allergies who developed aHUS after receiving an mRNA vaccine against SARS-CoV-2. She experienced symptoms like scleral bleeding, acute renal insufficiency, anemia, and thrombocytopenia. Despite initial treatment with plasma exchanges, remission only occurred after starting eculizumab therapy. Genetic testing revealed multiple inherited risk factors contributing to her condition.

Another case report documents a rare occurrence of acute pancreatitis in a pregnant woman following vaccination with the Pfizer-BioNTech mRNA vaccine for COVID-19 [27]. The patient, a 24-year-old South-Asian female at 31 weeks of gestation, presented with severe epigastric pain, nausea, and vomiting. She was diagnosed with acute pancreatitis, with no identifiable cause other than recent vaccination. Despite conservative management, including a spontaneous vaginal delivery and neonatal intensive care for the baby, the patient was discharged in a stable condition.

Last, Bennett et al. discussed a case of immune thrombocytopenia (ITP) in a pregnant patient following the initiation of the COVID-19 vaccination series [13]. The patient, in her first trimester, developed ITP 13 days after starting the vaccination. A thorough evaluation, including consultation with hematologists, confirmed the diagnosis. High-dose oral corticosteroids were administered, resulting in significant improvement in platelet count, and the patient was discharged home without complications. The report advocates for including pregnant women in clinical trials, emphasizing that the benefits of COVID-19 vaccination outweigh the risk of infection in pregnancy. It also suggests closer surveillance following vaccination in pregnant individuals until further data are available.

## 4. Discussion

The rise of vaccines in fighting COVID-19 has played a crucial role in diminishing adverse outcomes in various populations. Despite the clear benefits of vaccination, concerns regarding its safety in pregnant individuals have driven hesitancy within this demographic. In this comprehensive review, we synthesized findings from numerous studies to evaluate the safety and efficacy of COVID-19 vaccination in pregnant women.

The collective evidence from these studies underscores the safety and effectiveness of COVID-19 vaccination in pregnant and lactating individuals. Not only do vaccines elicit a robust immune response in pregnant individuals, but they also facilitate the transfer of vaccine-induced antibodies to the infant through cord blood and breast milk. Significantly, most studies have shown no link between COVID-19 vaccination and negative birth outcomes or newborn fatalities. The reported adverse effects have been mostly mild, such as pain at the injection site and fatigue. A recent multi-center cross-sectional study underscored the linear relationship between neutralizing antibody levels and duration post-vaccination in pregnant women, with mild to moderate adverse events observed, mirroring pre-pregnancy vaccination incidences [37]. Notably, inactivated vaccines demonstrated favorable immune persistence and safety profiles during pregnancy [37].

Numerous studies, particularly from 2021 onwards, have offered comprehensive insights into COVID-19 vaccination among pregnant women and newborns, emphasizing the lack of adverse outcomes. These studies extensively analyzed various factors, including preterm birth, stillbirth, birth weight, neonatal intensive care unit admission, neonatal death, Apgar score, injection-site pain, fever, headache, and fatigue. The literature seems to confirm the absence of severe adverse outcomes in pregnant women after vaccination [14,24,25,53,69] or the occurrence of mild ones, such as fever, following COVID-19 vaccination among pregnant women [29,38,43,48,49,62,71]. Further research focused on inactivated COVID-19 vaccines suggests that they were not associated with adverse pregnancy or neonatal outcomes [30,52]. Also, other studies indicated that neither the maternal inflammatory response nor the presence of SARS-CoV-2 antibodies in maternal blood had detrimental effects on the development of STB. Further reassurance regarding the safety of COVID-19 vaccination is emerging from several other studies, in which no severe adverse outcome was observed in the vaccinated pregnant population [22,41,51,57,70].

Furthermore, here we highlight the safety profiles of various types of COVID-19 vaccines, including inactivated vaccines and mRNA vaccines, irrespective of the timing and dosage of administration. While one study identified a potential trend toward an increased risk of miscarriage, overall evidence supports the safety of mRNA vaccines concerning the risk of miscarriages and congenital anomalies. However, the case reports and studies discussed above underscore the importance of closely monitoring adverse events following COVID-19 vaccination in pregnant individuals especially for potential associations with conditions such as fetal tachycardia and immune-mediated diseases. While raising concerns about potential risks, they also emphasize the overall benefits of vaccination in pregnancy, highlighting the need for ongoing research and surveillance to ensure the safety and efficacy of vaccination in the pregnant population [13,27,34,60].

This review acknowledges several limitations in the existing literature that warrant careful consideration. Notably, some studies included relatively small sample sizes, which may compromise the reliability and generalizability of their findings. Additionally, the review primarily relied on retrospective cohort studies, which are susceptible to selection bias. Furthermore, there was substantial heterogeneity in the populations examined across the included studies, with some failing even to specify the COVID-19 vaccine formulations used. Finally, the racial and ethnic composition of the study cohorts is an important factor that should be considered when interpreting the results. The findings of this review associated with the safety and efficacy profile of COVID-19 vaccination in pregnant and lactating populations, align with conclusions drawn from previous reviews and meta-analyses on the safety of vaccination [81,82,83] contributing valuable insights for public health strategies.

## 5. Conclusions

The evidence presented in this review strongly supports the recommendation for COVID-19 vaccination in pregnant women as a safe and effective strategy against the virus. Nonetheless, ongoing vigilance and continued monitoring of vaccine safety are crucial, particularly in light of emerging reports of adverse events. Ultimately, staying abreast of the evolving literature is imperative to ensure the well-being of pregnant individuals and their offspring in the context of COVID-19 vaccination.

## Figures and Tables

**Table 1:** Criteria- matched studies.

Study	Type of Vaccination	Type of Study
[10]	Pfizer-BioNTech, BNT162b2, Moderna, mRNA-1273 AstraZeneca, AZD1222	CS (retrospective)
[11]	Pfizer-BioNTech, BNT162b2	CaS
[12]	mRNA vaccination	CS
[13]	NA	CaS
[14]	NA	CS
[15]	Pfizer-BioNTech, BNT162b2	CS (prospective)
[16]	mRNA vaccination	CS (retrospective)
[17]	Pfizer-BioNTech, BNT162b2	OS
[18]	mRNA vaccination	CS
[19]	Pfizer-BioNTech, BNT162b2 AstraZeneca, AZD1222 -Pfizer-BioNTech, BNT162b2 CoronaVac, Sinovac -Pfizer-BioNTech, BNT162b2	RT (prospective)
[20]	mRNA vaccination	OS (retrospective)
[21]	mRNA vaccination	PCS
[22]	Pfizer-BioNTech, BNT162b2	CS (retrospective)
[23]	NA	CS
[24]	NA	CS (retrospective)
[25]	Johnson & Johnson, Janssen Pfizer-BioNTech, BNT162b2 Moderna, mRNA-1273	CS (retrospective)
[26]	mRNA vaccination	CS
[27]	Pfizer-BioNTech, BNT162b2	CaS
[28]	mRNA vaccination	CS (retrospective)
[29]	NA	CS (retrospective)
[30]	BBIBP-CorV, Sinopharm CoronaVac, Sinovac	CS (retrospective)
[31]	Johnson & Johnson, Janssen Pfizer-BioNTech, BNT162b2 Moderna, mRNA-1273	OS
[32]	NA	CS (retrospective)
[33]	AstraZeneca, AZD1222 Johnson & Johnson, Janssen Moderna, mRNA-1273 Pfizer-BioNTech, BNT162b2	OS
[34]	NA	OS
[35]	Pfizer-BioNTech, BNT162b2	CS (retrospective)
[36]	Pfizer-BioNTech, BNT162b2	OS
[37]	BBIBP-CorV, Sinopharm CoronaVac, Sinovac	OS
[38]	AstraZeneca, AZD1222 Bharat Biotech, Covaxin	OS
[39]	Pfizer-BioNTech, BNT162b2	CS
[40]	mRNA vaccination	OS
[41]	NA	OS
[42]	BBIBP-CorV, Sinopharm	CS (retrospective)
[43]	NA	CS (retrospective)
[44]	mRNA vaccination	CS (retrospective)
[45]	Johnson & Johnson, Janssen Pfizer-BioNTech, BNT162b2 Moderna, mRNA-1273	CS
[46]	Pfizer-BioNTech, BNT162b2 Moderna, mRNA-1273 Johnson & Johnson, Janssen	CS (prospective)
[47]	NA	OS
[48]	NA	OS
[49]	Pfizer-BioNTech, BNT162b2	CS (retrospective)
[50]	CoronaVac, Sinovac	CS (prospective)
[51]	mRNA vaccination	CS
[52]	NA	CS (prospective)
[53]	Pfizer-BioNTech, BNT162b2 Moderna, mRNA-1273 AstraZeneca, AZD1222	OS
[54]	mRNA vaccination	CS (prospective)
[55]	NA	OS
[56]	mRNA vaccination	CS
[57]	Pfizer-BioNTech, BNT162b2	CS (retrospective)
[58]	mRNA vaccination	CS (retrospective)
[59]	Pfizer-BioNTech, BNT162b2	CS
[60]	mRNA vaccination	CaS
[61]	NA	SCS (retrospective)
[62]	CoronaVac, Sinovac Pfizer-BioNTech, BNT162b2	CS (retrospective)
[63]	Johnson & Johnson, Janssen Moderna, mRNA-1273 Pfizer-BioNTech, BNT162b2	CS (observational)
[64]	Pfizer-BioNTech, BNT162b2	CS (prospective)
[65]	mRNA vaccination	DS
[66]	Pfizer-BioNTech, BNT162b2	In vitro study
[67]	NA	OS
[68]	mRNA, adenovirus-vectored, and unspecified vaccination	CS
[69]	AstraZeneca, AZD1222 Pfizer-BioNTech, BNT162b2 Moderna, mRNA-1273, Johnson & Johnson, Janssen	DS
[70]	Pfizer-BioNTech, BNT162b2	CS (retrospective)
[71]	CoronaVac, Sinovac Moderna, mRNA-1273 Pfizer-BioNTech, BNT162b2 AstraZeneca, AZD1222	DS
[72]	NA	CS

CS: cohort study; CaS: case study; OS: observational study; DS: descriptive study; SCS: single-center study; RT: randomized trial; NA: not available.

## Data Availability

The raw data supporting the conclusions of this article will be made available by the authors upon request.

## Supplementary Materials

The following supporting information can be downloaded at: https://www.mdpi.com/article/10.3390/diagnostics14161775/s1, Table S1: PRISMA 2020 checklist. Reference [9] is cited in the Supplementary Materials.
